# Factors influencing the implementation and uptake of a discharge care bundle for patients with acute exacerbation of chronic obstructive pulmonary disease: a qualitative focus group study

**DOI:** 10.1186/s43058-020-00017-5

**Published:** 2020-08-21

**Authors:** Marta Michas, Lesly Deuchar, Richard Leigh, Mohit Bhutani, Brian H. Rowe, Michael K. Stickland, Maria B. Ospina, for the COPD PRIHS-2 Group, Ron Damant, Irvin Mayers, Jerry Hall, Lee Green, Charles Yan, Sachin Pendharkar, Chris Mody, Stephen Field, Brandie Walker, Tara Lohman, Michael Roman, Jim Graham, Peter Farris, Allan Ryan, Kelly Mrklas, Roberta Dubois

**Affiliations:** 1 Division of Pulmonary Medicine, Faculty of Medicine & Dentistry, University of Alberta, Edmonton, Alberta Canada; 2 Alberta Health Services, Edmonton, Alberta Canada; 3 Section of Respiratory Medicine, Cumming School of Medicine, University of Calgary, Calgary, Alberta Canada; 4 Department of Emergency Medicine, Faculty of Medicine & Dentistry, University of Alberta, Edmonton, Alberta Canada; 5 School of Public Health, University of Alberta, Edmonton, Alberta Canada; 6 Department of Obstetrics & Gynecology and Medicine, Faculty of Medicine & Dentistry, University of Alberta, 220B Heritage Medical Research Centre, Edmonton, Alberta T6G 2S2 Canada

**Keywords:** Chronic obstructive pulmonary disease, COPD, Discharge care bundle, Discharge pathways, Clinical pathways, Focus groups, Qualitative research

## Abstract

**Background:**

Chronic obstructive pulmonary disease (COPD) is one of the most common causes of mortality and morbidity in high-income countries. In addition to the high costs of initial hospitalization, COPD patients frequently return to the emergency department (ED) and are readmitted to hospital within 30 days of discharge. A COPD acute care discharge care bundle focused on optimizing care for patients with an acute exacerbation of COPD has been shown to reduce ED revisits and hospital readmissions. The aim of this study was to explore and understand factors influencing implementation and uptake of COPD discharge care bundle items in acute care facilities from the perspective of health care providers and patients.

**Methods:**

Qualitative methodology was adopted. Nine focus groups were conducted using a semi-structured guide: seven with acute and primary/community health care providers and two with patients/family members. Focus groups were audiotaped, transcribed verbatim, and coded and analyzed using a thematic approach.

**Results:**

Forty-six health care providers and 14 patients/family members participated in the focus groups. Health care providers and patients identified four factors that can challenge the implementation of COPD discharge care bundles: process of care complexities, human capacity in care settings, communication and engagement, and attitudes and perceptions towards change. Both health care providers and patients recognized process of care complexity as the most important determinant of the COPD discharge bundle uptake. Processes of care complexity include patient activities in seeking and receiving care, as well as practitioner activities in making a diagnosis and recommending or implementing treatment. Important issues linked to human capacity in care settings included time constraints, high patient volume, and limited staffing. Communication during transitions in care across settings and patient engagement were also broadly discussed. Other important issues were linked to patients’, providers’, and system attitudes towards change and level of involvement in COPD discharge bundle implementation.

**Conclusions:**

Complexities in the process of care were perceived as the most important determinant of COPD discharge bundle implementation. Early engagement of health providers and patients in the uptake of COPD discharge bundle items as well as clear communication between acute and post-acute settings can contribute positively to bundle uptake and implementation success.

Contributions to the literature

- Research has shown that discharge bundles improve chronic obstructive pulmonary disease (COPD) outcomes.
- This research identified challenges associated with implementation and uptake of care bundles at discharge from acute care from the perspective of patients and providers.
- Complexity of processes of care is perceived as the most important determinant of COPD discharge bundle uptake in acute care clinical settings from the perspective of patients and providers.
- The research results permit providers to tailor strategies to mitigate these challenges.

## Background

Chronic obstructive pulmonary disease (COPD) is one of the most common causes of mortality and morbidity in Canada and worldwide [1–3]. An important part of the health care burden of treating individuals living with COPD involves the management of acute exacerbations (AECOPD) [4] which become more frequent with disease progression. COPD is one of the most common reasons for hospitalization in Canada [5]. In addition, individuals living with COPD are the most common group to return to the emergency department (ED), and the largest group of patients readmitted to hospital within 30 days of discharge [6].

Care bundles, defined as “a small set of evidence-based interventions for a defined patient population and segment/care setting that, when implemented together, will result in significantly better outcomes than when implemented individually” [7], have been shown to improve patient outcomes when implemented in hospital settings for diverse patient populations, particularly in critical care of mechanically- ventilated patients [8, 9]. Discharge care bundles in COPD focus on optimizing care for patients with an AECOPD as they transition from acute care settings back to the community. In a systematic review examining the effectiveness of COPD discharge care bundles [10], evidence suggested that these discharge bundles may reduce ED revisits and hospital readmissions.

Although the idea of discharge care bundles seems straightforward, their successful implementation in clinical settings can be challenging [11] and often requires a tailored strategy that maximizes patient and clinician engagement. Further, sustaining adoption within the clinical setting is a unique challenge, with limited proven strategies to ensure long-term uptake. While the emergence of evidence in dissemination and implementation science provides tools and strategies for the design of practice improvements [12], challenges in bringing these same improvements to frontline clinicians and patients and subsequently sustaining the adoption of these improvements in clinical settings are addressed less often. As COPD discharge bundles have been developed to facilitate transitions of care, their evaluations within trials have shown that actual uptake can be very low [13], necessitating a better understanding of implementation barriers and strategies [10, 14]. The existing literature on implementation of discharge bundles for patients with COPD exacerbations is very limited, and knowledge gaps remain regarding the challenges associated with implementing discharge care bundles. Even more limited is the perspective of the patients and practitioners on the barriers and facilitators impacting uptake of the intervention. This manuscript uses the voice of patients and providers to contribute evidence to a common challenge in a field where a paucity of evidence is available. The aim of this study was to explore the perspectives of patients and health care providers about factors influencing successful implementation of COPD discharge care bundles in acute care hospitals. This study will inform and support the development of implementation strategies designed to improve transitions of care from acute care settings into the community.

## Methods

### Study design

This qualitative study using a focus group methodology is part of a broader project aimed at developing, implementing, and evaluating a discharge care bundle for individuals with AECOPD admitted to acute inpatient and ED settings in Alberta (Canada). Briefly, the broader project was planned to develop a COPD discharge bundle (as part of an end-to-end pathway) to be implemented in both ED and acute inpatient settings across the province, and evaluate its effectiveness in reducing ED and hospital readmissions and improve patient-centered and economic outcomes. Details of care bundle development are described elsewhere [10, 15] and resulted in a COPD discharge care bundle that includes seven interventions: (1) ensure patient has demonstrated adequate inhaler technique, (2) send discharge summary to family physician office and arrange follow-up, (3) optimize and reconcile respiratory medications, (4) provide a written discharge management plan and assess patient and care giver comprehension of discharge instructions, (5) refer to pulmonary rehabilitation, (6) screen for frailty and comorbid condition(s), and (7) assess smoking cessation readiness, provide counseling, and refer to smoking cessation program, where appropriate. Consensus was used to finalize the content of the evidence-based COPD discharge bundle, and prior to its evaluation via a stepped-wedge trial (ClinicalTrials.gov identifier: NCT03358771), this qualitative research was conducted as part of the knowledge translation strategy to inform provincial implementation [16].

### Study setting and participants

Focus groups were conducted in acute and community/primary care settings from large urban (Edmonton, Calgary), moderate-sized regional (Red Deer), and rural (Slave Lake) centers in Alberta (Canada) between October 2015 and February 2016 to seek input from both patients with COPD and health care providers. Ethics approval was obtained from the University of Alberta Research Ethics Board (Pro00055500); written informed consent to participate in the study was obtained from participants.

Nine focus groups were conducted; segmentation was defined a priori, with seven focus groups being conducted with health care providers and two with patients. The recruitment aimed for between four and eight participants for each session; this size is suggested to be ideal for non-commercial focus groups [17]. The study used purposive sampling to recruit participants. Health care participants for the focus groups were initially invited through communication with unit managers and clinical leads in acute care sites, administrative managers in primary care settings, and direct email invitation sent to frontline staff. Health care participants that were contacted directly were encouraged to invite their eligible colleagues. Eligible health care participants included clinical staff caring for patients with COPD and administrative staff from inpatient and ED units (acute care) or locations (primary care) that admit and/or care for patients with COPD. The focus groups were conducted during regular working hours, and remuneration for participation was not provided; therefore, it was essential to establish a good rapport with clinical and administrative leaders in each setting so they would approve the 1-h commitment for this research activity, where the study team provided lunch or snacks.

Patient participants were recruited from two regular and well-established patient support groups, one in a large urban center (Edmonton) and one in a regional center (Red Deer). In order to reduce the burden of travel for patients, the support group leaders were asked for permission to have the research team participate in one of their regular weekly meetings and conduct the focus group there. Participants from large urban locations were individuals living with COPD who had recently graduated from the pulmonary rehabilitation (PR) program at the local lung center, and patients from the regional center were long-term members of a support group that included patients with various lung diseases. For practical reasons, patients were not enrolled in rural health care facilities due to the lack of availability of research staff and well-established patient support groups in these settings.

### Data collection

The focus groups were moderated by experienced (MSc and PhD-level) female research facilitators and members of the research team (MM, LD). None of the focus group facilitators were directly involved with the care of patients with COPD nor knew any of the study participants prior to the focus groups. The focus group sessions lasted for 1 h. Sessions were audio recorded and transcribed verbatim by a note taker or research coordinator. Additional field notes from each team member documented reflections during the focus group sessions. Field notes were used to supplement the focus group findings during the thematic analysis.

Each focus group opened with a short overview presentation about the context of the study and background on how the various components of the project interdigitated. The facilitator highlighted that their information was being used to potentially help patients and health care providers improve care processes for the management of acute COPD exacerbations. Health care providers were offered a copy of the presentation slides, while patients were offered a plain language informal conversation to provide an overview of the project. In addition, all participants were provided with a written plan-language summary included in the consent forms. The focus groups were conducted with the aid of a semi-structured focus group guide (Additional file 1) developed according to the Theoretical Domains Framework (TDF) [18]. Briefly, the TDF is a comprehensive framework of theoretical domains including individual (rational, cognitive, emotional) and external, organizational variables (working environment, resources) that influence behavioral change and innovation uptake. The TDF has been widely used to inform implementation of interventions in respiratory research and clinical practice [19–21].

### Data analysis

Focus group verbatim transcripts were coded using the NVivo 11 qualitative analysis software [22] and prepared for thematic analysis. Thematic analysis is a foundational method of qualitative analysis for identifying, organizing, describing, and reporting themes found within qualitative data [23, 24]. This analytical approach is a useful method for examining the perspectives of different research participants (i.e., health providers and patients) [24, 25]. Three researchers (MM, LD, MBO) analyzed transcripts independently to identify and classify emerging themes. Two of these researchers read the transcripts and coded them line by line using an iterative approach. Codes with similar meaning were grouped together to form categories, which later were compared and merged into larger themes. We used the criteria suggested by Krueger and others [26, 27] as a framework for interpreting coded data: words, context, internal consistency, frequency and extensiveness of comments, specificity, and intensity of comments. Theoretical saturation was reached when no additional data was found to develop additional themes in the data analysis [28]. Researchers met to discuss the thematic categories that emerged from the data, with discrepancies being independently resolved by a third researcher (MBO). Field notes were used to triangulate the results and supplement the focus group findings. Descriptive statistics were used to report extensiveness (coding coverage, defined as percentage of time spent on discussions about the identified themes). We used the Consolidated Criteria for Reporting Qualitative Research (COREQ) [29] in the reporting (see Additional file 2).

## Results

### Characteristics of participants

Seven focus groups with health care providers (*n* = 46) and two focus groups with patients (*n* = 14) were conducted; the group sizes varied from 5 to 11 participants. Abridged characteristics of the participating health care providers and patients are provided in Tables 1 and 2, respectively.

**Table 1:** Characteristics of 46 health providers participating in the qualitative study on implementation of COPD care bundles

	Acute care settings (*N* = 35), *n* (%)	Community and primary care settings (*N* = 11), *n* (%)
Sex		
Male	17 (49)	2 (19)
Female	18 (51)	9 (81)
Age		
Under 29	2 (6)	–
30 to 39	9 (26)	2 (19)
40 to 49	8 (23)	6 (55)
50 to 59	13 (38)	1 (10)
60 and older	3 (9)	2 (19)
Clinical discipline*		
Medical		
Respirologist	7 (20)	–
Emergency physician	4 (12)	–
Family physician/general practitioner	–	2 (19)
Pharmacist	–	1 (10)
Allied and administrative		
Respiratory therapist	7 (20)	–
Physical therapist	–	–
Primary care nurse	3 (9)	2 (19)
Occupational therapist	–	1 (10)
Nurse practitioner	2 (6)	2 (19)
Unit manager	3 (9)	–
Clinical nurse educator	4 (12)	–
Registered nurse	4 (12)	–
Social worker	1 (3)	–
Licensed practical nurse	–	1 (10)
Clinical assistant	1 (3)	–
Administrative positions	1 (3)	2 (19)
Work setting*		
Acute care hospital	26 (75)	–
Emergency department	14 (40)	–
Specialist clinic	5 (15)	–
Primary care—family practice clinic	–	8 (73)
Primary care—walk-in clinic	–	2 (19)
Other		
Management	–	2 (19)
Specialist physician administrative office	1 (3)	–
Years of practice		
< 2	2 (6)	–
2 to 5	4 (12)	3 (28)
6 to 10	6 (18)	–
11 to 15	4 (12)	1 (10)
16 to 20	3 (9)	–
20 to 25	3 (9)	3 (28)
26 to 30	4 (12)	2 (19)
> 30	9 (26)	2 (19)
Location of practice		
Urban	35 (100)	
Regional	–	6 (54)
Rural	–	5 (46)

*NR* not reported

*Multiple responses were allowed

**Table 2:** Characteristics of 14 patients participating in the qualitative study on the implementation of COPD care bundles

	Total (*N* = 14), *n* (%)
Sex	
Male	8 (57)
Female	6 (43)
Age	
50 to 59	1 (7)
60 and older	13 (93)
Place of residence	
Small town	1 (7)
Urban	13 (93)
Living conditions*	
Live independent	3 (21)
Live with someone who helps with care	10 (71)
Drive to appointments	12 (86)
Rely on others to go to appointments	6 (43)
Type of lung condition*	
Patients with COPD**	10 (71)
Other lung condition (*N* = 8)***	5 (63)
Time since COPD diagnosis (years)	
< 2	1 (7)
2 to 5	3 (21)
6 to 10	4 (29)
11 to 15	2 (14)
16 to 20	2 (14)
Number of hospital visits due to lung condition within last year	
0	10 (71)
1	3 (21)
2–3	1 (7)
Preferred location of health care*	
Large urban	8 (57)
Regional	8 (57)
Small town	1 (7)

*NR* not reported

*Multiple responses were allowed

**Question only asked to patients in focus group #9; all patients in focus group #5 were pulmonary rehabilitation program graduates and had COPD diagnosed at the admission

***Question asked only to the participants of focus group #9

#### Health care providers

We aimed to interview a broad spectrum of health care providers from various locations, clinical settings, and professions (Table 1). Acute care providers were located in tertiary care facilities in two large urban settings, while primary health care providers were located in one regional and one rural primary care setting where the number of patients would be less; however, the potential for stronger relationships between providers and between providers and patients exists. Other settings included specialist clinics, walk-in clinics, and managerial/administrative areas.

The most represented clinical providers included respirologists and registered respiratory therapists, although all nursing roles combined (educators, administrators, and other non-bedside nursing roles) represented the highest proportion of participants. The analysis for all acute care participants (inpatient and ED) was combined into one group, and the analysis for primary/community care participants was combined into another group. In all further discussion, the following three units of analysis were used: acute care health providers, primary care/community health care providers, and patients.

#### Patients

Of the 14 participants in the patient/family member focus groups (Table 2), the majority lived with someone who could help them with their health care needs and were able to drive themselves to their medical appointments. While the majority of the patients participating in the focus groups had a diagnosis of COPD, several were unsure of their lung health diagnosis. Of the patients who were able to reflect on acute care admissions for AECOPD, all reported shortcomings in their perception of the management of one or more of their hospital stays and openly shared where they saw or had heard of better ways to manage AECOPD.

All participants in the focus group conducted in a large urban location had a confirmed COPD diagnosis as it was a qualifying condition for the participation in the pulmonary rehabilitation program; however, not all patients knew that the term applied to them. For the second focus group involving a lung support group operating in a smaller urban area, patients had various lung conditions; therefore, an additional question was added to the questionnaire (see “Type of lung condition” field in Table 2). As this research relates to exploring barriers and facilitators to uptake of and adherence to clinical support guidelines and tools, the information this latter population shared was considered relevant to the study.

### Factors influencing implementation

**Fig. 1:**
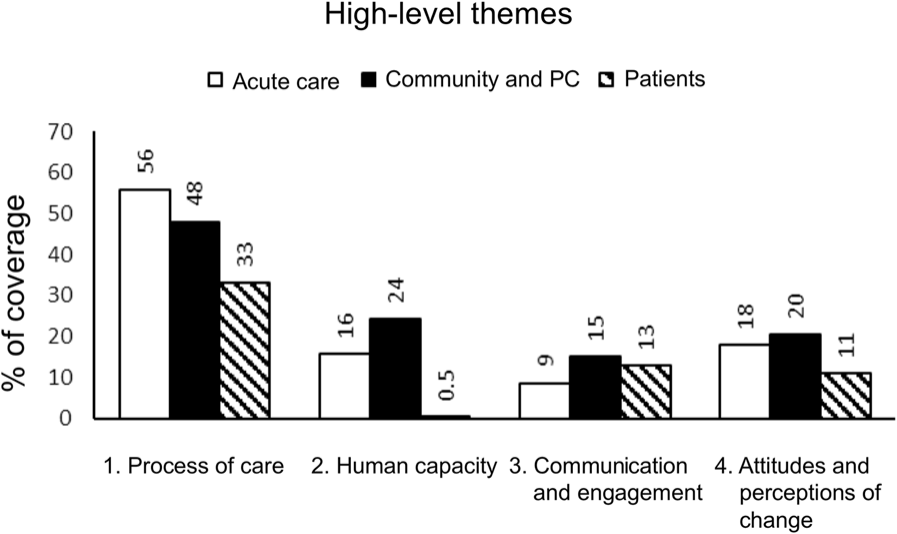
Coverage of high-level themes during the focus group. Themes are not mutually exclusive

**Fig. 2:**
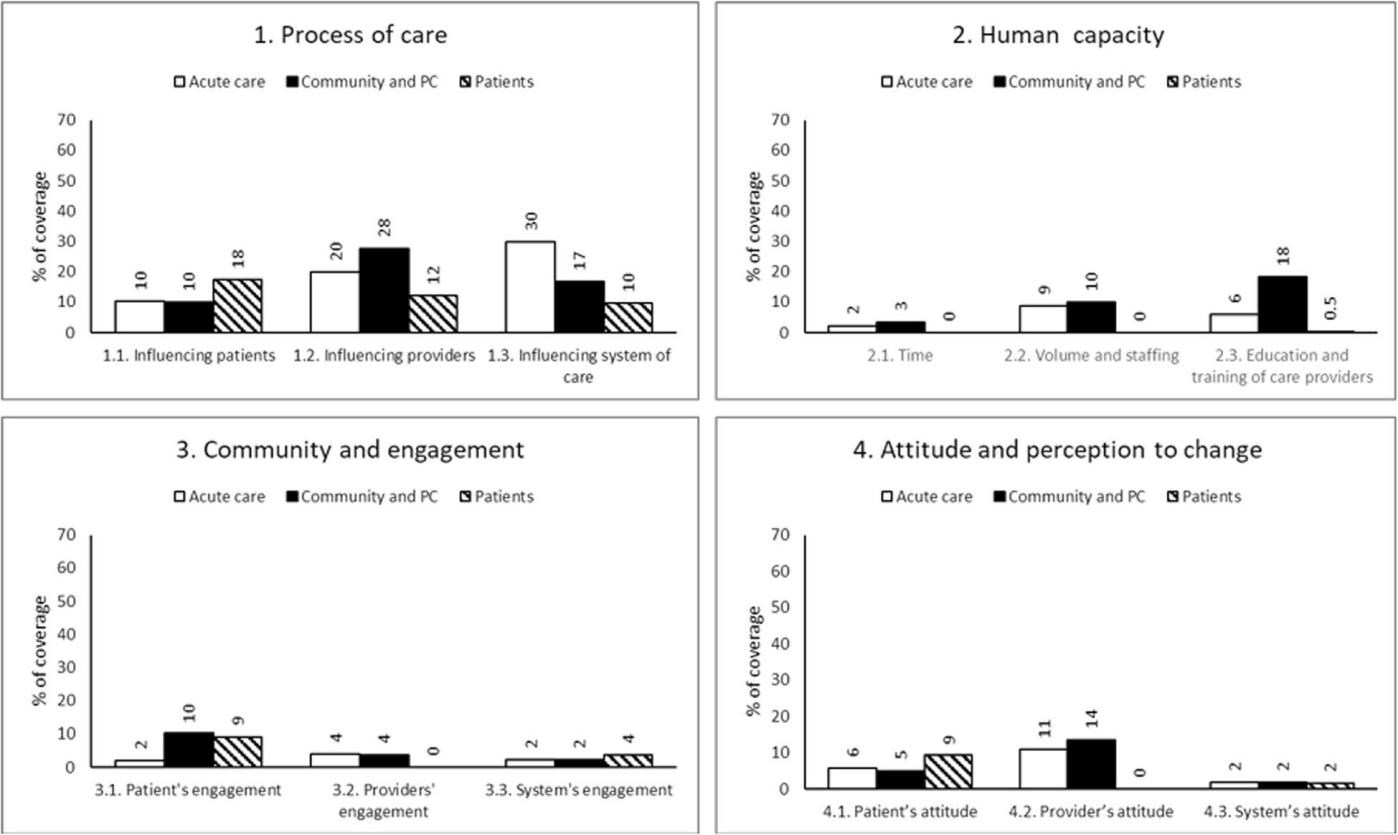
Coverage of sub-themes during the focus groups. Sub-themes are not mutually exclusive. **1** Process of care. **2** Human capacity. **3** Community and engagement. **4** Attitude and perception to change

The analysis identified four emerging themes influencing COPD discharge care bundle implementation as discussed by participants: (1) process of care, (2) human capacity in care setting, (3) communication and engagement, and (4) attitudes and perceptions towards change. These high-level themes were further divided into sub-categories (Table 3). Figures 1 and 2 provide a descriptive summary of the themes and extensiveness (percentage of time spent on discussions about the identified themes) among health care provider and patient focus groups, respectively.

**Table 3:** Description of themes used for focus groups coding

Theme	Description
1. Process of care	Definition: “What is actually done in giving and receiving care. It includes the patient’s activities in seeking care and carrying it out as well as the practitioner’s activities in making a diagnosis and recommending or implementing treatment” [30]
1.1. Influencing patients	Example: cost of medications and access to services from the patient perspective
1.2. Influencing providers	Example: not sure who is doing what, challenges with patient diagnosis, patient transfer to another unit
1.3. Influencing care system	Example: lack or presence of pulmonary rehabilitation services, access to pharmacist, lack or presence of family doctor
2. Human capacity in care setting	Definition: the ability of the people implementing the discharge care bundle items (nurses, RRTs) to make sure the items are attended to
2.1. Time constraints	Example: not enough nursing/RRT staff time to implement additional steps in care, patients do not spend enough time in ED
2.2. Volume and staffing issues	Example: nursing/RRT staff shortage
2.3. Education and training of health care providers	Example: training of staff on inhaler techniques, training of staff on discharge care bundle
3. Communication and engagement	Definition: the level of engagement and communication within single setting (such as buy-in) or across specializations (such as acute primary care)
3.1. Patients’ engagement	Example: communication between patient and provider, patient engagement/interest in self-managing, information overload
3.2 Providers’ engagement	Example: buy-in from frontline/physicians
3.3. System’s engagement	Example: communication and collaboration across sites, multidisciplinary communication and collaboration
4. Attitude and perception of change	Definition: set of psychological/administrative responses to planned change. This includes positive and negative responses
4.1. Patient attitudes	Example: do not want to do new things/willingness to do so, opinion that the intervention is not worth the effort
4.2. Provider attitudes	Example: opinion that intervention not useful, attitude towards checklist—positive and negative
4.3. System attitudes	Example: administrative obstacles, support from executive management

*ED* emergency department, *RRT* registered respiratory therapist

#### Theme 1: Process of care

The process of care theme was defined as “what is actually done in giving and receiving care.” It includes patient activities related to seeking care and carrying out care instructions, as well as practitioner activities related to making a diagnosis and recommending or implementing treatment [31]. Processes of care were further sub-categorized into those influencing patients (e.g., cost of medications), process of care influencing providers (e.g., uncertainty of roles when executing discharge care bundle), and process of care influencing the care system (e.g., access to pharmacists in the acute care setting). Process of care was the theme most frequently discussed by all participant groups.

Patients spent the majority of the time discussing *process of care influencing patients*. Some of these processes of care mentioned by patients included operational activities that, albeit not attributable to specific clinical interventions, they influence patients’ uptake of COPD discharge bundle implementations (e.g., waiting times, ability and support to book follow-up appointments, and connect with other level of care). Some patients discussed the level of education they received from health care providers in both acute care and community settings as an important aspect of processes of care for patients, while others commented on the potential positive influence of a patient management plan or checklist as part of a discharge care bundle on patient outcomes:

Patient1: you used to get lots of explanations, how and what (...) what I find is that you don’t get that anymore. Patient2: if you ask for it you get it. You have to ask for it yourself. Facilitator: so, would that be helpful if you had a checklist, so you knew the things you had to have? Patient2: possibly, yes (...) Patient3: that would help.

Health care providers also acknowledged the challenges patients are facing when accessing health care services, particularly in rural communities. Challenges articulated could also be related to the confusion described among team members and roles:

Primary health care provider: they travel, each one of them at the moment. We would have the person that covers [few rural communities] here (...) But then if you take [few other communities] (...) that area doesn’t have an RT (...) So they travel

For processes of care influencing health care providers, acute care providers often discussed which members of the provider team should be responsible for executing discharge care bundle items (which varied across sites represented); the proper allocation of the tasks to qualified providers was seen as a challenge in the implementation process:

Acute care provider: [we don’t know] (...) if it was nurse initiated actions, physician initiated actions, care coordinator or pharmacy, you know, the people involved in our area.

Primary and community health care providers’ comments were process-focused, and their discussion of their own role in the implementation of care bundle items indicated a lack of continuity and provider/program linkage in the system as it serves patients currently:

Primary health care provider: So, my first reaction (...) was “where do we, as a [Primary Care Network] PCN, fit in this?” Because this looks like (...) these (...) interventions are all sort of done in the emergency department or in hospital (...) if this care bundle is being done by someone in hospital, so where are we going to fit in to make sure this care bundle is put in the place that’s already being done in acute care setting.

Primary health care providers also noticed challenges in caring for patients with multiple comorbidities—a common problem with COPD patients and a common problem in primary care, when the need of the patient is surrounding the coordination of up to five subspecialty clinics/providers.

Primary health care provider: (...) [patient] is in ER and you have all the comorbidities, the way we are working at the moment we are going diagnosis specific. So the same nurse discharging the patient has a checklist for heart failure, for COPD, for asthma and for how to manage the bone and joint.

Access to a registered respiratory therapist, pulmonary rehabilitation program (particularly in rural communities), lack of attachment to family doctors, and transitions in care were some of the subjects widely discussed under process of care influencing care system.

Primary health care provider: (…) in 2012, 85% of our [rural] communities do not have the correct level of RT to sustain the services provided at that facility. So, even if we double or triple what we had, I can still not guarantee that they will be at the pulmonary rehab level

Primary health care provider: So, they might not have a family doctor (… ),, they might not have an easy access to them, [and] what kind of communication is coming from the hospital to the family doctor and their team to alert them that they can bring this patient in soon enough.

#### Theme 2: Human capacity in care settings

Human capacity in care settings was defined as the ability of the people directly engaged in the implementation (e.g., nurses, registered respiratory therapists) to ensure that the individual care bundle items are executed. Human capacity was further divided into three sub-categories: time constraints related to the implementation (e.g., not enough time available to spend with each patient), volume and staffing issues (e.g., nursing shortages), and education and training of health care providers (e.g., sufficient or insufficient knowledge of the proper use of a discharge care bundle).

Human capacity in care setting was discussed more often among the community and primary health care providers than the acute care providers (which could be related to setting), while aspects related to the qualifications of health care providers gained the most interest among patient focus group participants.

Primary health care provider: (...) in emergency departments and hospitals, it is the nurses the ones who are doing a big education piece. They are not going to provide as good teaching as they could if they don’t understand the disease process and a treatment for it.

Issues related to volume and staffing were rarely mentioned in both health care provider groups, except when it came to respiratory therapists, at which time the lack of access to a registered respiratory therapist was often mentioned among both acute care and primary health care provider participants.

Facilitator: (...) your [emergency] nurses and docs [are] too busy to do [discharge care bundle], but there is that opportunity for respiratory therapists? Acute care provider: (...) there’s two for the entire department, both paediatrics and adults and there should be 3-4 full time (...)

Time required (to implement/uptake new practices) was minimally discussed among acute care providers and community and primary health care providers. These provider groups, however, recognized that implementation of the discharge care bundle would require additional time. Patients did not identify and discuss issues related to human capacity in care settings*.*

#### Theme 3: Communication and engagement

The communication and engagement theme was described as the level of engagement and communication to facilitate transitions in care within each setting (e.g., buy-in from frontline staff—provider engagement), across specializations (e.g., acute primary care communications—system’s engagement), and individual patient’s interest in self-management (patient engagement).

Patient engagement was the most discussed sub-theme among both community and primary health care providers and patients (Fig. 2, panel 3); however, this subject was seldom discussed by acute care providers. Community and primary health care providers primarily talked about the need to increase patient compliance, potentially through engagement in the care bundle implementation across programs and services:

Primary health care provider: Most people (...) as soon as their cough starts to improve they are not going to stop (therapies). They realize this is how it works, this is why it does that, I have to continue for it to keep working. (A comment regarding the probability that once people see an effect from their therapies, they are more likely to continue using the therapy).

Patients often commented about the importance of engagement in self-management and importance of communication and establishing a relationship with both primary and specialist health care providers after hospital or ED discharge:

Patient: Our doctor said if we are going South this year he would give us a care package and it would have a couple of antibiotics and five days of prednisone so if [we] did get sick we could get home, with the prednisone.

*Provider engagement* and *system engagement* received a small amount of attention from any of the participants, with the most significant coverage (4%) of *provider engagement* among acute care providers, and relating to the degree of attachment that patients had as well as the degree to which the patients were engaged in the bundle elements (education as an example).

#### Theme 4: Attitudes and perceptions of change

This theme was characterized by a set of psychological responses to planned change, comprised of both positive and negative participant responses to potential discharge care bundle implementation, such as patients’ willingness or refusal to try new things, staff’s positive or negative attitude towards checklist, or support or lack thereof from site management.

Among both groups of health care providers, *provider’s attitude* was the most commonly discussed sub-theme (Fig. 2, panel 4) within the *attitudes and perception of change* high-level theme. Patients also discussed *patient’s attitude*; however, *system’s attitude* was discussed less frequently in all focus groups.

Acute care providers recognized that buy-in from their colleagues would be an important factor influencing discharge care bundle uptake and implementation. Some providers were involved in unsuccessful implementations in the past and discussed skepticism regarding the current effort, or expressed negative attitude towards checklists/bundles in general, based on past negative experiences.

Acute care provider: No offense though, I’m chasing physicians down left, right and center, so them filling out that paper? They're not going to fill that out.

In general, primary health care providers recognized the importance of being involved in the COPD pathway implementation effort but also pointed out there are competing projects that often restrict their time to participate wholly on one project for one system or disease, when their patients are often living with comorbid and complex health issues.

Primary health care provider: (...) this is only the second or third pathway coming down the pipeline, there is another hundred coming.

Patients expressed their willingness to follow recommendations, instructions, or referrals provided to them at or before discharge from the ED or hospital, based on their previous experiences being discharged from an acute care setting for COPD care.

## Discussion

This study provides insights about factors influencing implementation and use of a COPD discharge care bundle in acute care facilities from the perspective of health care providers and patients. Health care providers and patients in all focus groups identified complexities in process of care as the most important factor potentially affecting implementation and subsequent uptake of the COPD discharge care bundle. Acute care providers reflected mostly on system processes, while community and primary health care provider discussions reflected more on the impact on providers. Patients concentrated mostly on how the process of care influenced themselves, such as lack of educational information, lack of access to pulmonary rehabilitation programs, or how difficulties in accessing family doctors may negatively impact the implementation and uptake of the discharge care bundle. Patients commented on how the process of care led to positive beliefs about the benefits of instructions related to disease management given to them before discharge. The complexity of process of care is perceived as very context-driven and refers to tasks roles, continuity through referrals, and education. Depending on the number of provider tasks, programs, and services available, the level of complexity in the processes of care is amplified.

Community and primary care providers and patients recognized patient engagement as having an impact on implementation and uptake of the discharge bundle; health care providers commented on engaging with patients to ensure better compliance while patients discussed the importance of self-management and engaging with health care providers after discharge. This common focus on patient engagement is a clear signal to implementation planners that shared decision-making between patients and providers across the care continuum has the potential to positively impact implementation and uptake of care bundles in acute care settings. Interestingly, acute care providers did not see patient engagement having an impact on bundle implementation; with bundle initiation occurring in the acute care setting, the opportunity to improve the patient experience across the continuum of care is limited.

Attitudes and perception of change were discussed in all study groups. Attitudes towards changes in practice were most often discussed sub-themes among health care providers and patients, respectively. From the provider perspective, ensuring proper buy-in from the frontline staff was recognized as a facilitator to implementation, although achieving this engagement in light of competing responsibilities may result in a negative attitude towards the intervention and was consequently perceived as a barrier. Employees who are overwhelmed by parallel initiatives may show symptoms of change fatigue and would passively accept the imposed changes but not engage in the process [32, 33] putting the implementation at risk of failure. It is worth noting that no symptoms of change resistance (actively criticizing and denying the need for a change [32, 34]) were registered during the focus groups. Primary and community health care providers had challenges in recognizing where they fit in the whole process; they seemed to be discouraged by the sense of disconnection, which was also echoed in the discussion about buy-in and attitude to change. In general, system attitude, defined as a set of psychological/administrative responses to planned change (e.g., administrative obstacles or support from executive management), were not seen as important factors for implementation, suggesting the forces influencing uptake were perceived to be related mostly to the frontline staff directly involved in the discharge care bundle execution. These results align with research conducted by Khodyakov et al. [35] on factors related to adoption of a surgical site infection prevention bundle, in which physician buy-in was perceived as a very important determinant of bundle implementation. In their case, however, staff resistance was also manifested, contrary to observations made during this study.

Time allocated to the implementation of the bundle was not seen as an important factor to successful implementation by all study groups. Issues related to work volume and staffing, however, were noted by both acute and community and primary health care providers as a barrier to uptake. An example includes the lack of access to respiratory therapist which could negatively impact completion of several bundle elements (e.g., inhaler technique teaching and smoking cessation counseling).

While community and primary health care providers saw education and training as a more vital contributor to uptake, they did not describe lack of time as a barrier. Contrary to our findings, Lennox et al. [36] found lack of time (“staff too busy”) as the most prominent barrier to bundle implementation in their study on implementation of a COPD care bundle in hospitals in London (UK). However, similar to our study results, they recognized staff shortages, staff engagement, and added workload among the top four barriers to bundle implementation, barriers that the most robust implementation plan would be challenged with.

Our study showed that health care providers appreciate the importance and benefits of the COPD discharge care bundle. The results suggest a benefit of early engagement of health providers in the implementation process, as some of the care bundle items require communication between acute and post-acute settings. Engaging primary, community, and acute care providers directly involved in the COPD discharge care bundle execution is an important aspect of uptake and sustainability of the change. While ideal, given the spread of providers in a community, at times accomplishing this goal may be difficult. Moreover, patient engagement is necessary as some of the proposed items are associated with patient compliance. Although, in this study, patients did not directly focus on barriers and facilitators for the discharge bundle implementation in the acute care settings, their comments on how the intervention affects them can be valuable when planning bundle implementation. The patients reflected on their own ability to impact outcomes, more than the role of the providers in either acute or primary care community. Some evidence suggests that patient engagement and understanding of the implementation process may change staff behavior and attitude towards implementation and uptake through informed patients requesting staff to perform specific actions [37, 38].

There are some noteworthy limitations to this study. First, there is the question of the generalizability. As participation in the focus group was voluntary, it is possible that acute and primary health care providers who volunteered were those who are most likely early adopters and more willing to engage in the early conversations and facilitate implementation. The health providers were experienced and older, and these results may not apply to younger or less-experienced health providers. Health care focus group participants may not properly represent opinions of late adopters or those hesitant to change. For the patient participants, all patients were relatively healthy and were not housebound, and while most had experience with a COPD hospitalization, most were infrequent users of the acute care system for COPD-related care. As patient participants were recruited from well-established patient support groups and some were graduates from PR programs, it is likely that their level of knowledge, engagement, and compliance differs from other patients discharged with COPD that have not accessed these programs. Further, the patients sampled had access to specialists (pulmonologists/respirologists), were attached to primary health care providers, and were participating in pulmonary rehabilitation programs or support groups in large urban and regional health centers. This group does not likely represent all individuals living with more advanced COPD, those living in rural areas, or who are not well connected to primary or specialist providers. Second, the study was conducted prior to finalization and implementation of a provincial standardized COPD discharge care bundle. Third, neither providers nor patients had experience with COPD care bundles in practice, so experience in employing the tool could not be used to further inform the research. Future research involving those with experience with the bundle is required for iterative revisions. Finally, inpatient and ED health care providers were combined in one unit of analysis (“acute care providers”). The authors appreciate there are differences between inpatient and ED providers, their time constraints, and the granular level of their knowledge about COPD. There are also differences between urban and rural settings; however, as the focus of the work was for participants to inform barriers and facilitators from their perspective, the authors felt they could aggregate inpatient and emergency feedback.

## Conclusions

By identifying clinically relevant factors influencing implementation and use of the COPD discharge care bundle perceived by patients and health care providers, this study built an understanding of the challenges potentially associated with implementation and uptake of the said bundle in acute care settings. Participants recognized complexity of process of care (“what is actually done in giving and receiving care”), including the patient’s activities in seeking care and carrying it out (“as well as the practitioner’s activities in making a diagnosis and recommending or implementing treatment” [31]), as the most important determinant of the COPD discharge bundle adoption. Other important issues were linked to human capacity in care settings (time constraints, volume and staffing, education and training of health providers); issues with engagement of patients, providers, and the health care system; and patients’, providers’, and system attitude and perception of change. The knowledge gained in this study will allow planning of strategies tailored to address and mitigate specific challenges and to stimulate successful bundle implementation and uptake.

## Supplementary information

**Additional file 1.** Semi-structured focus group guide on Identification of care gaps, barriers and facilitators for the implementation COPD care bundle in hospital and ED settings in Alberta.

**Additional file 2.** Consolidated criteria for reporting qualitative studies (COREQ): 32-item checklist.

## Data Availability

The data supporting the findings of this study are available upon request from the corresponding author (MBO). The data are not publicly available due to containing information that could compromise research participant privacy.

## Abbreviations

AECOPD: Acute exacerbation of chronic obstructive pulmonary disease
AHS: Alberta Health Services
COPD: Chronic obstructive pulmonary disease
ED: Emergency department
ILC: Innovation Learning Collaborative
PCN: Primary Care Network
PR: Pulmonary rehabilitation
RT: Respiratory therapist
SCN: Strategic Clinical Network™
