# Supplementary material for: Factors influencing the implementation and uptake of a discharge care bundle for patients with acute exacerbation of chronic obstructive pulmonary disease: a qualitative focus group study
Source: Implement Sci Commun. 2020 Aug 21;1:3. doi: 10.1186/s43058-020-00017-5 (PMC7477849; doi:10.1186/s43058-020-00017-5)
Supplement: Supplementary file 1 — Additional file 1. Semi-structured focus group guide on Identification of care gaps, barriers and facilitators for the implementation COPD care bundle in hospital and ED settings in Alberta. [file 43058_2020_17_MOESM1_ESM.docx]

**Additional File 1.** Semi-structured focus group guide on Identification of care gaps, barriers and facilitators for the implementation COPD care bundle in hospital and ED settings in Alberta

**Focus Groups Topics Guide for Health Care Providers**

| Knowledge and Skills | Characteristics of COPD patients’ journey  Care gaps  What happens when the patient leaves the emergency room  What happens when the patient admitted to the hospital  What happens when the patient leaves the hospital |
| --- | --- |
| Nature of the Behaviours | What are your thoughts on the content? Is there anything that needs to come off? Is there anything you feel should be on and is not? Was the language easy to understand? What are your thoughts on the format? (explore different options) |
| Decision Processes | What happens when a patient with a COPD flare up is admitted to the emergency room?  Opinions about different treatment recommendations after emergency/hospital discharge |
| Social/Professional Role and Identity | Would you feel comfortable with using the checklist? Do you think there is a need for training? (if yes, explore who would need training, how and where?) |
| Environmental Context and Resources | Preferred channels to receive the pathway.  How would you make it easier to use/implement?  What are the main issues around actually using the checklist here?  What are the barriers to using the checklist? What are the enablers? |
| Beliefs about Capabilities | How comfortable you would be to follow the recommendation of the bundle? |
| Social Influences | What do you think you and other staff in your practice would react towards the check list? (What would people think/say/do?) |
| Emotion | What would drive the positive/negative reaction towards implementing the bundle? If negative, how could it be rectified?  How is your sense of comfort in following the recommendations of the pathway |
| Beliefs About Consequences | What do you think about the aims of having the Checklist as a one page reminder of important things one must not forget prior to discharge from hospital/ED? (explore efficiency, teamwork, communication, transitions in care)  Do you think the checklist is likely to improve outcomes of COPD patients? If not, why not? |
| Motivation and Goals | What do you think about checklists that doctors and patients can use to guide the COPD treatment after emergency and hospital leave.  What can prevent this list from being used in emergency rooms and hospitals. |
| Behavioural Regulation | When we start implementation of the bundle, what do you think will be the biggest obstacle? What would help? |

**Focus Groups Topics Guide for Patients**

| Emotion | What do participants have in mind when they think about the treatment of COPD |
| --- | --- |
| Beliefs about Capabilities | Opinions about the recommendations that health providers give at emergency or hospital discharge. |
| Environmental Context and Resources | Perception of the current care that patients of COPD receive after they have been in the emergency room or hospital |
